# Oral Supplementation With a Bovine Thymus Extract Reduces Neuronal Excitability in Aging Mice

**DOI:** 10.1096/fba.2025-00256

**Published:** 2026-01-30

**Authors:** Abdeslem El‐Idrissi, Natalia Surzenko, Bassem F. El‐Khodor

**Affiliations:** ^1^ Department of Biology and Center for Developmental Neuroscience, College of Staten Island The City University of New York New York New York USA; ^2^ Biology Program, The Graduate Center The City University of New York New York New York USA; ^3^ Nutrition Innovation Center Standard Process Inc Kannapolis North Carolina USA

**Keywords:** EEG, GABA, neuronal excitability, thymus extract

## Abstract

Gamma‐aminobutyric acid (GABA) is the primary inhibitory neurotransmitter in the central nervous system (CNS). Many aspects of GABAergic neurotransmission, including the densities of GABAergic neurons, the synthesis of GABA and its interaction with the respective receptors, are believed to be altered during aging, contributing to increased neuronal excitability seen in multiple neurodegenerative conditions, such as dementias, Alzheimer's disease, and traumatic brain injury (TBI). Oral administration of a nuclear fraction extract of the bovine thymus gland (thymus nuclear fraction—TNF) to rats was recently reported to improve their functional recovery from controlled cortical impact (CCI)—an animal model of TBI. Given that individual thymic peptides and mixed thymus fractions were also found to have broad neuroprotective effects and anti‐neuroinflammatory activity, we sought to investigate the impact of TNF on GABAergic neurotransmission in the aging mouse brain. Using biochemical investigation, electrophysiological recordings, obtained using electroencephalography (EEG), and power spectral density analysis, we evaluated GABAergic protein expression and cortical neuronal activity in aged control mice and in mice supplemented with a low dose (LD) or a high dose of TNF for 14 weeks. We uncovered increased expression of two isoforms of glutamic acid decarboxylase, GAD65 and GAD67, and increased levels of β2/β3 subunits of GABA_A_ receptor in the brains of TNF‐supplemented mice compared to the control group, suggesting possible enhancement of inhibitory neurotransmission. Decreased neuronal excitability, evidenced by reduced EEG amplitudes, power spectral densities, and peak amplitudes of high‐frequency cortical oscillations, further confirmed a dose‐dependent attenuation of neuronal excitability by TNF. Our results suggest that TNF supplementation may have the potential to mitigate age‐related alterations in GABAergic neurotransmission, thereby modulating neuronal excitability.

## Graphical abstract

Oral supplementation of aging mice with a nuclear fraction extract of bovine thymus (TNF) leads to significant increases in GAD65, GAD67, and β2/β3 subunits of the GABA_A_ receptor, more so with high dose (HD) TNF treatment compared to low dose (LD) and control condition. Increases in GABAergic protein expression are accompanied by better locomotor activity in HD TNF‐supplemented animals. Supplementation with TNF extract reduces cortical excitability in a dose‐dependent manner. Created in https://BioRender.com.

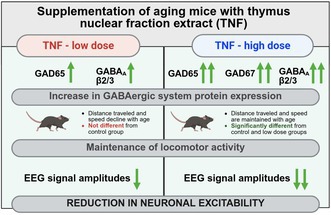

## 1 Introduction

Aging of the brain is accompanied by a range of neurochemical changes that perturb the functions of structural proteins, neuropeptides, neurotransmitters, and their related receptors, along with increasing oxidative stress and neuroinflammation [1, 2, 3]. Together, the age‐related neurochemical shifts in the brain lead to a multitude of pathological sequelae, starting from aberrant intercellular signaling and ending with cell loss, tissue atrophy, and the progressive functional deterioration that manifests in declining memory performance, poor coordination of movements, and worsening sensory processing [4].

One of the key neurotransmitters whose function is affected by aging is gamma‐aminobutyric acid (GABA), which primarily mediates the inhibitory neurotransmission throughout the central nervous system (CNS) [5]. The GABAergic system encompasses the biosynthesis and metabolic degradation of GABA, its release from neurons, interactions with the respective receptors, and its inactivation in GABAergic and glutamatergic neurons and in astrocytes [6]. Various aspects of the GABAergic system change as we age [7, 8, 9, 10, 11, 12]. For instance, multiple studies have observed a significant decline in GABA receptors in the brains of older human adults (> 55 years of age) and older experimental animals [9, 13, 14, 15]. Additionally, the levels of the enzyme that is crucial for GABA synthesis, glutamic acid decarboxylase (GAD), specifically its two isoforms, GAD65 and GAD67, significantly decrease in the cortex and hippocampus in aged compared to young rats [16, 17, 18]. Finally, the reduction in the numbers of GABAergic neurons themselves may greatly impact age‐related disruption of inhibitory neurotransmission [10, 11, 15, 19, 20, 21, 22].

Consistent with the compromised GABAergic signaling during aging, multiple age‐related neurologic diseases, such as Alzheimer's disease and dementias, are accompanied by the increased cortical neuronal excitability [10, 23]. Moreover, dysfunction of the GABAergic system, along with the elevated neuronal excitability, is considered a main contributor to the pathophysiology of Alzheimer's disease, as well as to the prevalence of epileptic seizures among older individuals [10, 24, 25, 26, 27, 28].

Similarly, the long‐lasting pathological consequences of traumatic brain injury (TBI) also involve dysregulation of the GABAergic neurotransmission, while posttraumatic epilepsy is one of the devastating latent outcomes of TBI [29, 30, 31, 32]. Loss of GABA‐mediated neuroprotection following injury and during aging may further exacerbate TBI pathology and outcomes, evidenced by the increased risks of chronic traumatic encephalopathy (CTE), a progressive neurodegenerative disease, and Alzheimer's disease following TBI [33, 34, 35]. In fact, many changes that take place in the aging brain may resemble TBI pathology and include neuroinflammation, axonal degeneration, mitochondrial dysfunction, excitotoxicity, oxidative stress, cellular senescence and apoptotic cell death of neurons and glia [36, 37, 38]. Together, these pathological processes underlie the onset and the progression of the neurodegenerative disease during aging and following brain trauma and can also accelerate the process of brain aging itself [37, 38, 39].

Nutritional support is considered essential for the maintenance of brain health during aging and following TBI, and has been linked to the modulation of the GABAergic system [6, 40, 41, 42]. Furthermore, disease‐modifying therapies that are derived from natural sources are highly desirable, as they carry low risk of systemic toxicity. A recent study has demonstrated that a nutritional supplement containing a nuclear fraction extract of the bovine thymus gland (Thymus Nuclear Fraction, TNF) enhances the functional recovery of rats from controlled cortical impact (CCI) injury—an animal model of TBI [43]. Thymus nuclear fraction (TNF)‐mediated behavioral improvements include accelerated restoration of animal vestibulomotor and cognitive functions following CCI and are accompanied by the changes in the expression of hippocampal genes involved in the modulation of neuroinflammation and tissue repair.

Thymus gland arises from neuroectoderm, and in addition to its well‐known primary role in the development of T cells that are vital for the adaptive immunity, can serve neuromodulatory and anti‐inflammatory functions through the production of hormone‐like peptides [44, 45, 46]. Emerging evidence suggests that thymus‐derived extracts and peptides, including thymosin, thymopoietin, thymopentin, thymulin and other mixed thymic preparations, may have a broad impact on the nervous system and can enhance neuroprotection and regeneration, while reducing neuroinflammation [43, 44, 46, 47, 48, 49, 50].

In this study, we aimed to test TNF, which was found to be beneficial for the recovery from TBI [43], for its potential effectiveness in regulating the inhibitory GABAergic neurotransmission in the aging brain—a system that recapitulates many aspects of TBI and neurologic disease. Our study results reveal that oral administration of TNF to aged mice increases the expression of the proteins that serve essential functions in the GABAergic system and attenuates age‐related increase in neuronal excitability, measured using electroencephalography (EEG), thereby providing insight into possible avenues for the modulation of inhibitory neurotransmission during aging.

## 2 Materials and Methods

### 2.1 Animals

A total of 45 mice were used in this study. Adult male C57BL/6 mice (12 months of age) were purchased from Charles River Laboratories (RRID:MGI:7264769; Wilmington, MA, USA). Because the first evidence for TNF's potential to modulate CNS function came from studies conducted in males and needed further validation, female mice were not included in the current study [43]. Animals were received at the vivarium of the College of Staten Island/City University of New York (CUNY) and assigned unique identification numbers. Animals were housed individually in ventilated cages and were acclimated for 7 days prior to initiation of the study. All mice were examined, handled, and weighed prior to the study initiation to assure adequate health and suitability. During the study, 12/12 h light/dark cycles were maintained. The room temperature was maintained between 20°C and 23°C with a relative humidity around 50%. Chow (Laboratory Rodent Diet 5001; Cat.#0001319; LabDiet, St. Louis, MO, USA) and water were provided ad libitum for the duration of the study. Animals were randomly assigned across treatment groups. Body weights were taken twice a week during the study. Table 1 shows the experimental design and the timing of the procedures. All procedures were approved by the Institutional Animal Care and Use Committee (IACUC) in accordance with the National Institute of Health Guide for the Care and Use of Laboratory Animals (IACUC # 20–005).

**TABLE 1:** Experimental design.

Description	Weeks (W)			
	W(−1)	W1	W8	W12
Vehicle		Oral gavage, two times per day		
TNF—low dose (LD)				
TNF—high dose (HD)				
Body weight	Twice weekly			
Acclimation	X			
Open field test		X	X	
EEG recordings				X
Terminal tissue collection				X

### 2.2 Thymus Nuclear Fraction (TNF) Supplement

The nutritional supplement was provided by Standard Process Inc. (Palmyra, WI, USA). Nuclear extracts were made from frozen raw bovine glands and dried on‐site using Standard Process Inc. proprietary processes. The product is in powder form and is suitable for consumption by animals and humans. This extract has been commercially available as a dietary supplement in the United States since the early 1950s.

The powder was homogeneously dispersed in sterile water and administered by oral gavage twice a day at a dose volume of 3.5 mL/kg. Fifteen mice were used in each treatment group. Group 1: treatment at low dose of 170 mg/kg twice a day (340 mg/kg/day); Group 2: treatment at high dose of 340 mg/kg twice a day (680 mg/kg/day); Group 3: vehicle control (water administered at a dose volume of 3.5 mg/kg).

### 2.3 Western Blot

At the end of the study, mice were sacrificed by decapitation and brains were immediately dissected within 1 min, frozen on dry ice and stored at −80°C. Total soluble and membrane bound proteins were extracted [51] and protein concentration was determined using the Pierce BCA Protein Assay Kit (Thermo Fisher, Rockford, IL, USA). For GABA receptor β2 and β3 subunits detection, 15 μg of protein was loaded onto a Mini‐PROTEAN TGX 4%–16% gradient polyacrylamide‐SDS gel (Bio‐Rad Laboratories Inc) and transferred onto PVDF membrane (Millipore, Bedford, MA) after electrophoresis. Membranes were sequentially probed with two antibodies: a monoclonal antibody that recognizes β2 and β3 subunits of GABA_A_ receptor (Clone 62‐3G1; Millipore, Temecula, CA) and a rabbit polyclonal antibody (A2668, Sigma, St. Louis, MO) that recognizes β‐actin (1:10,000 dilution) as a control for protein loading. For GAD detection, 10 μg of protein was used, and membranes were simultaneously probed with two antibodies: SC‐365180, a monoclonal (1:5000 dilution, Santa Cruz Biotechnology, Santa Cruz, CA) that recognizes both GAD65 and GAD67 isoforms and anti β‐actin as above. After incubation with the primary antibodies overnight at 4°C, the membranes were washed with PBS‐T and incubated with a 1:5000 dilution of either goat anti‐mouse IgG (H + L) (IRDye 680, Thermo Fisher Scientific, Rockford, IL) or goat anti rabbit IgG (H + L) (IRDye 800, Thermo Fisher Scientific, Rockford, IL) secondary antibodies at 1:5000 for 1 h at room temperature with gentle shaking. Following the secondary antibody incubation, the membranes were washed with PBS‐T three times and then washed two times with 1XPBS. The membranes were imaged with an Odyssey infrared imaging system (LI‐COR Biosciences) at a scan intensity of 5 and resolution of 169 μm. Western blot images were quantified using the National Institutes of Health ImageJ version 1.53 software (Bethesda, MD, USA). The values for GABA_A_ receptor subunits and GAD levels were normalized to β‐actin, and density ratios compared to control values were calculated for each sample.

### 2.4 Open Field Test

The open field was constructed of a 62 cm by 62 cm laminated wood floor surrounded by clear acrylic walls of 30 cm height. A 4 × 4 grid matrix was painted on the floor of the open field, dividing the field into 16 squares (12 outer, 4 inner) each measuring 15.5 cm by 15.5 cm. The experimenter was blinded to mouse treatment. The light in the behavior testing room was maintained at 200 lx. At the beginning of the open field session, mice were placed on the right front corner square (relative to the position of the experimenter) and were retained inside a clear acrylic tube (diameter = 3.5 in.). After 30 s, the tube was lifted and the mouse was allowed to move freely about the open field for a total period of 5 min, then returned to its home cage. The open field was washed with Windex between each session. The behavioral parameter measured was the total number of grid crossings made during the 5‐min trial. A crossing was defined as all four paws moving out of one of the 16 squares and into another. This measure is expected to correlate highly with the distance traveled in the open field, and it reflects locomotor activity, exploratory behavior and anxiety levels. Open field sessions were videotaped, and animal performance was analyzed by an experimenter blinded to mouse treatment.

### 2.5 Intracerebral Recordings of Local Field Potentials

Mice were anesthetized with ketamine/xylazine mix (90/10 mg/kg i.p.), scalps were shaved, then fixed on a stereotaxic apparatus. Right side craniotomies were made at AP 2.5 mm from bregma, L 0.5 mm (medial prefrontal cortex). Extracellular recordings were obtained with tungsten electrodes with impedances of 1–2 MΩ. Electrodes were placed in infragranular layers (0.5 lateral and 1.0–1.2 mm deep in prefrontal cortex). Local field potential (LFP) from the prefrontal cortex was recorded. LabChart‐8 (AD Instruments, Colorado Springs, CO, USA) was used for LFP recording. This includes both—the frequency domain and time domain features that have been extracted. The low‐frequency oscillations (LFO): delta 0.4–4 Hz, theta 5–7 Hz, alpha 7–12 Hz, beta 13–25 Hz, gamma 26–80 Hz. High frequency oscillations (HFO): slow ripples 125–250 Hz and fast ripples 250–500 Hz. All recordings were passed through a preamplifier connected to the electrode and amplified using model 1700 differential AC Amplifier (AD Instruments) and digitized at 10 kHz.

### 2.6 Power Spectral Density Analysis

Electrophysiological signal is represented in the time domain; this signal can be processed using the Fast Fourier Transform (FFT) which converts the time domain signal to the frequency domain. In this way, we can examine the collected change of amplitude within a given sine wave. Since a frequency is composed of many sine waves, each wave varies in amplitude. Through the FFT, we can identify at which component of a frequency contains changes in energy produced in its amplitude. This is accomplished using the power spectral density (PSD) analysis. With this tool, we can represent the changes of energy produced within the frequency domain. This is used to identify the major frequencies that exist and to show the differences in the rate of oscillation of various frequencies and the amount of energy produced in each component.

### 2.7 Statistical Analysis

Statistical analysis was performed using IBM SPSS Statistics, version 29 and GraphPad Prism, version 10.4.2. Open field and body weight data were analyzed using Two‐Way Repeated Measures ANOVA and EEG data with Two‐Way ANOVA, followed by Tukey's Post Hoc test, where indicated. Peak amplitude of the response was calculated using LabChart software (AD Instruments, Colorado Springs, CO, USA). Western blot data were analyzed using One‐Way ANOVA and unpaired Student's *t*‐test. Significant difference was set at *p* < 0.05. Data were expressed as mean ± standard error of the means (S.E.M.).

## 3 Results

### 3.1 Thymus Extract Supplementation Increases the Levels of GABAergic System Proteins in the Brains of Aging Mice

**FIGURE 1:**
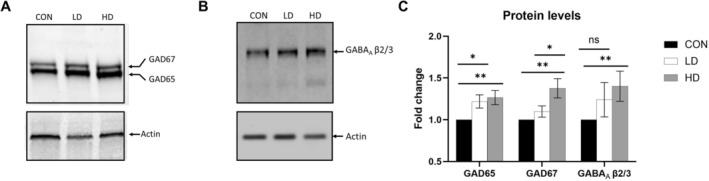
Expression of GABAergic markers in the brains of TNF‐supplemented mice. (A) Representative western blot of GAD65/67 and β‐Actin expression in the brains of control (CON) and TNF‐supplemented mice: LD–Low dose, HD–High dose. (B) Representative western blot of GABA_A_ β2/3 and β‐Actin expression in the brains of control mice (CON) and mice supplemented with TNF at a low dose (LD) or a high dose (HD). (C) Fold changes in GAD65/67 and GABA_A_ β2/3 protein levels in control (CON) and TNF‐supplemented LD and HD groups. GAD65 levels were significantly increased in TNF‐LD and TNF‐HD brains compared to control animals (*p* = 0.011 and *p* = 0.0045, respectively). No difference in GAD65 was detected between the LD and HD groups (*p* = 0.688). The levels of GAD67 were significantly increased in HD group animals compared to control group (*p* = 0.003). No significant differences were found between the CON and LD groups (*p* = 0.151), while GAD67 levels were increased in the HD compared to LD group (*p* = 0.048). Data are shown as means ± SEM; fold change for control samples was set to 1; *p ≤ 0.05; **p ≤ 0.005 by Two‐Way ANOVA and Tukey's post hoc test.

To begin investigating the potential of TNF to modulate GABAergic neurotransmission in the aging brain, we supplemented 12‐month‐old male mice with either a low (LD) or a high dose (HD) of TNF daily by oral gavage for the duration of 12 weeks. Brain levels of β2/β3 subunits of the GABA_A_ receptor and two isoforms of GAD enzyme (65 and 67) were measured using Western blot. GAD65/67 enzymes are essential for the synthesis of GABA in GABAergic neurons, while their expression levels in the brain decrease with age [11, 52, 53]. Here, we found that brain tissues of TNF‐supplemented aging mice contained significantly higher levels of GAD65 and GAD67, compared to control brains (GAD65: F[2,36] = 4.47, *p* = 0.018, partial eta squared = 0.20; GAD67: F[2,36] = 6.43, *p* = 0.004, partial eta squared = 0.263) (Figure 1A,C). Specifically, both LD and HD supplementation regimens increased GAD65 protein levels (*p* = 0.011 and *p* = 0.0045, respectively) compared to the control group, while no difference in GAD65 levels was detected between the LD and HD groups. On the other hand, GAD67 levels increased significantly only in the HD group animals compared to the control group (*p* = 0.003), while the LD supplementation regimen did not induce a significant increase in GAD67 expression compared to control animals, resulting in a significant difference in GAD67 between the LD and the HD groups (*p* = 0.048) (Figure 1A,C).

Ligand‐gated GABA_A_ receptor, including its key β2/β3 subunits, is responsible for the majority of fast inhibitory neurotransmission in vertebrates and its expression has been reported to be altered in the aging brain and in Alzheimer's disease [11, 54]. Protein levels of β2/β3 subunits of the GABA_A_ receptor were significantly increased in HD group animals compared to controls (*p* = 0.003), but were not significantly different between the control and LD (*p* = 0.064) or LD and HD groups (*p* = 0.569) (F[2,21] = 4.11, *p* = 0.031, partial eta squared = 0.28) (Figure 1B,C). These data suggest that TNF supplementation can preserve the expression of key molecular components of the GABAergic system in the aging rodent brain, which may in turn impact neurotransmission.

### 3.2 Locomotor Activity Is Preserved in Aging Mice Supplemented With TNF at High Dose

**FIGURE 2:**
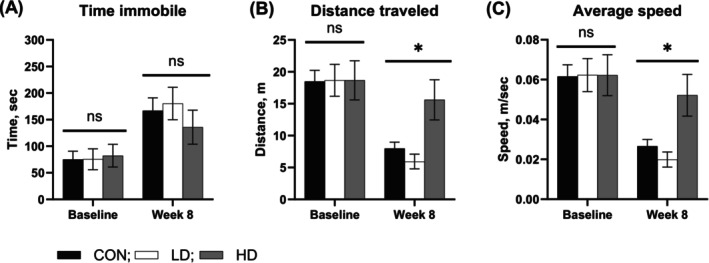
Locomotor activity on an open field test in control (CON) mice and mice supplemented with TNF at a low dose (LD) or a high dose (HD). (A) No significant changes were detected between the three groups in times spent immobile at baseline or at 8 weeks of supplementation. (B) Total distance traveled during a 5‐min test was not different between the groups at baseline but remained increased in HD group animals compared to CON and LD groups (*p* < 0.05) at week 8 of supplementation. (C) The average travel speed did not differ between the groups at baseline, but was significantly higher in HD compared to CON and LD group animals (*p* < 0.05). *—Significantly different from CON and LD, *p* < 0.05; ns—not significant by Two‐Way Repeated Measures ANOVA and Tukey's post hoc test.

**FIGURE 3:**
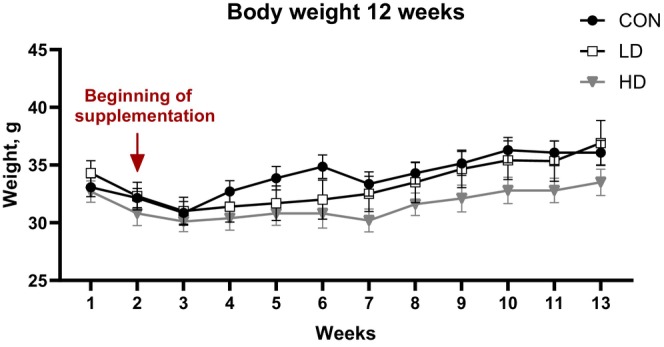
Changes in body weights of control (CON) mice and mice supplemented with TNF at a low dose (LD) or high dose (HD). Animals in the HD group displayed a trend towards reduction in average body weight during the supplementation period compared to both CON and LD mice.

The observed increases in the expression of GABAergic system proteins in aging TNF‐supplemented animals may suggest possible alterations in mouse behaviors related to alertness or locomotion [55, 56, 57]. We therefore examined the activity of control and TNF‐supplemented mice using an open field behavioral test. We found that the time spent immobile (the inverse of the time spent moving) did not significantly differ between the treatment groups at baseline or at week 8 of the study (Figure 2A). Two‐Way Repeated Measures ANOVA showed significant treatment × time interaction for total distance traveled (F[2,34] = 4.195, *p* = 0.0235, partial eta squared = 0.198) and average travel speed (F[2,34] = 4.185, *p* = 0.0237, partial eta squared = 0.192). Total distance traveled and the average travel speed did not differ between all three groups at baseline (Figure 2B,C). However, mice supplemented with TNF at a high dose (HD group) traveled significantly longer distances and maintained significantly higher travel speed at week 8 of supplementation compared to LD and control group animals (*p* < 0.05) (Figure 2B,C). Consistent with the maintenance of locomotor activity, we observed that HD group animals displayed a trend towards reduction in the average body weight compared to control and LD mice (Figure 3). Two‐Way Repeated Measures ANOVA showed significant treatment × time interaction for body weight (F[22,385] = 2.86, *p* < 0.0001, partial eta squared = 0.14). However, Tukey's post hoc test did not show a significant difference between the groups at any time. These results confirm that the increased expression of GAD65/67 and GABA_A_ receptor β2/β3 subunits in TNF treated animals, along with TNF‐induced reduction in the power of cortical oscillations across multiple bands discussed below, are not associated with a negative impact on the locomotor behavior of aging animals.

### 3.3 Thymus Extract Supplementation Reduces Neuronal Excitability

**FIGURE 4:**
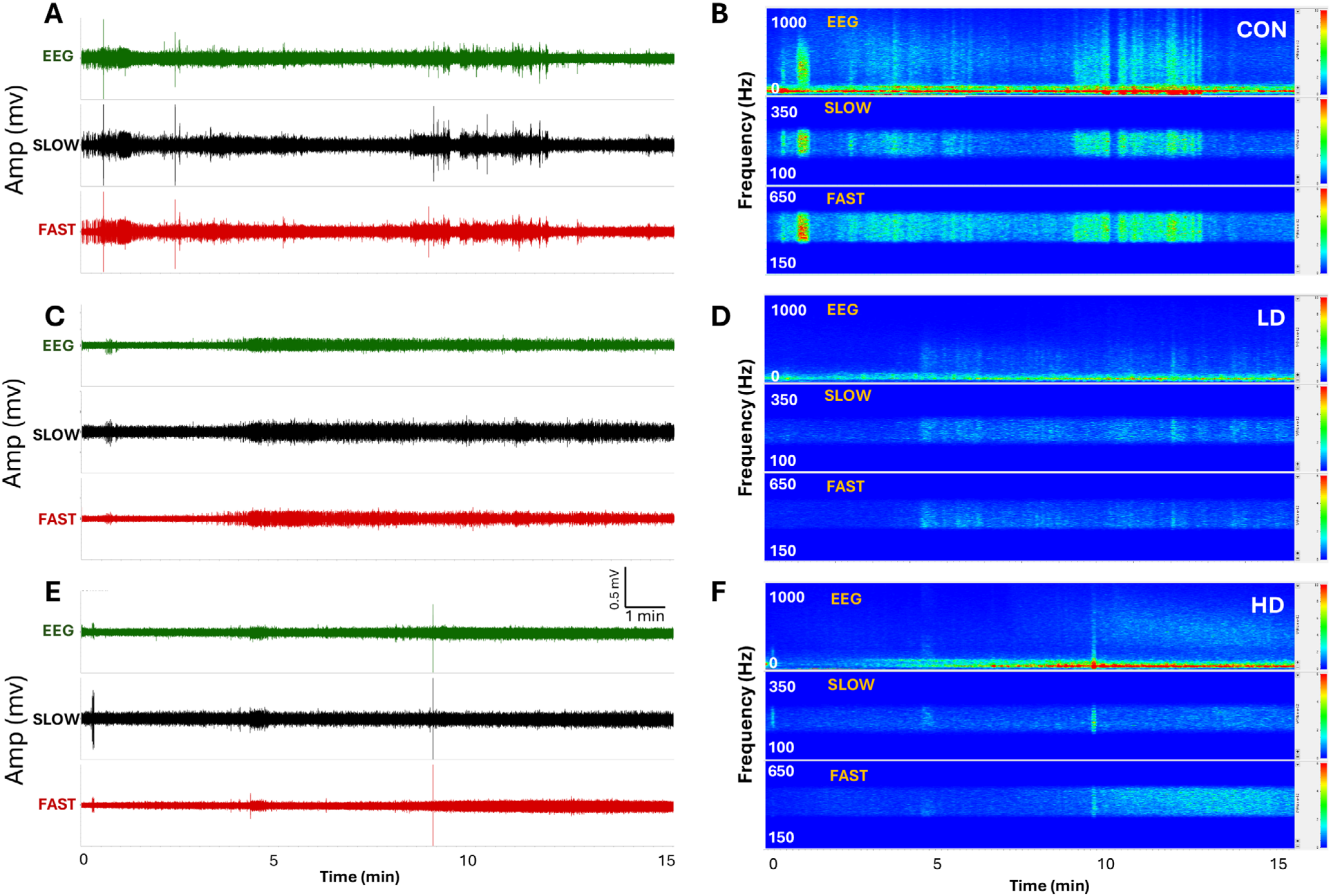
Representative recordings of field potentials in the prefrontal cortex. Spontaneous electrophysiological activity was measured in the prefrontal cortices of control mice (CON) and mice supplemented with TNF at a low‐dose (LD) or a high‐dose (HD) (A, C and E, upper traces). Field potentials were recorded for 15 min, and the resulting traces were filtered between 0.4 and 500 Hz and amplified. (A, C, E) The original traces (green—upper) and filtered traces (black—slow ripples 125–250 Hz; red—fast ripples 250–500 Hz). (B, D, F) The power densities of field potentials and filtered frequencies were converted into heat maps for control animals (B) and animals supplemented at a low dose (D) or a high dose (F).

To investigate whether TNF‐induced increase in the expression of proteins responsible for the synthesis of GABA and for GABAergic neurotransmission has an impact on the function of neuronal circuits, we first examined neuronal excitability in control and TNF‐supplemented mice by measuring cortical field potentials. Synaptic activity triggers membrane currents that pass through the extracellular space and are measured as local field potentials using the electrodes placed outside the neurons (Figure 4A,C,E, upper traces). Field potential traces were filtered between 0.4 and 500 Hz and amplified. The LFOs are defined as follows: delta 0.4–4 Hz, theta 5–7 Hz, alpha 7–12 Hz, beta 13–25 Hz, gamma 26–80 Hz. HFOs are slow ripples at 125–250 Hz and fast ripples at 250–500 Hz (Figure 4A,C,E, middle and lower traces). Conversion of electrophysiological recordings and high frequency oscillations into a heat map shows a significant decrease in the heat intensity spectra for animals supplemented with TNF extracts (Figure 4B,D,F) compared to controls. These results demonstrate a clear effect of TNF supplementation on neuronal excitability, evidenced by the decreases in the amplitudes of both—slow and fast ripple activities.

### 3.4 Thymus Extract Supplementation Suppresses the Amplitudes of the EEG Signals and PSDs

**FIGURE 5:**
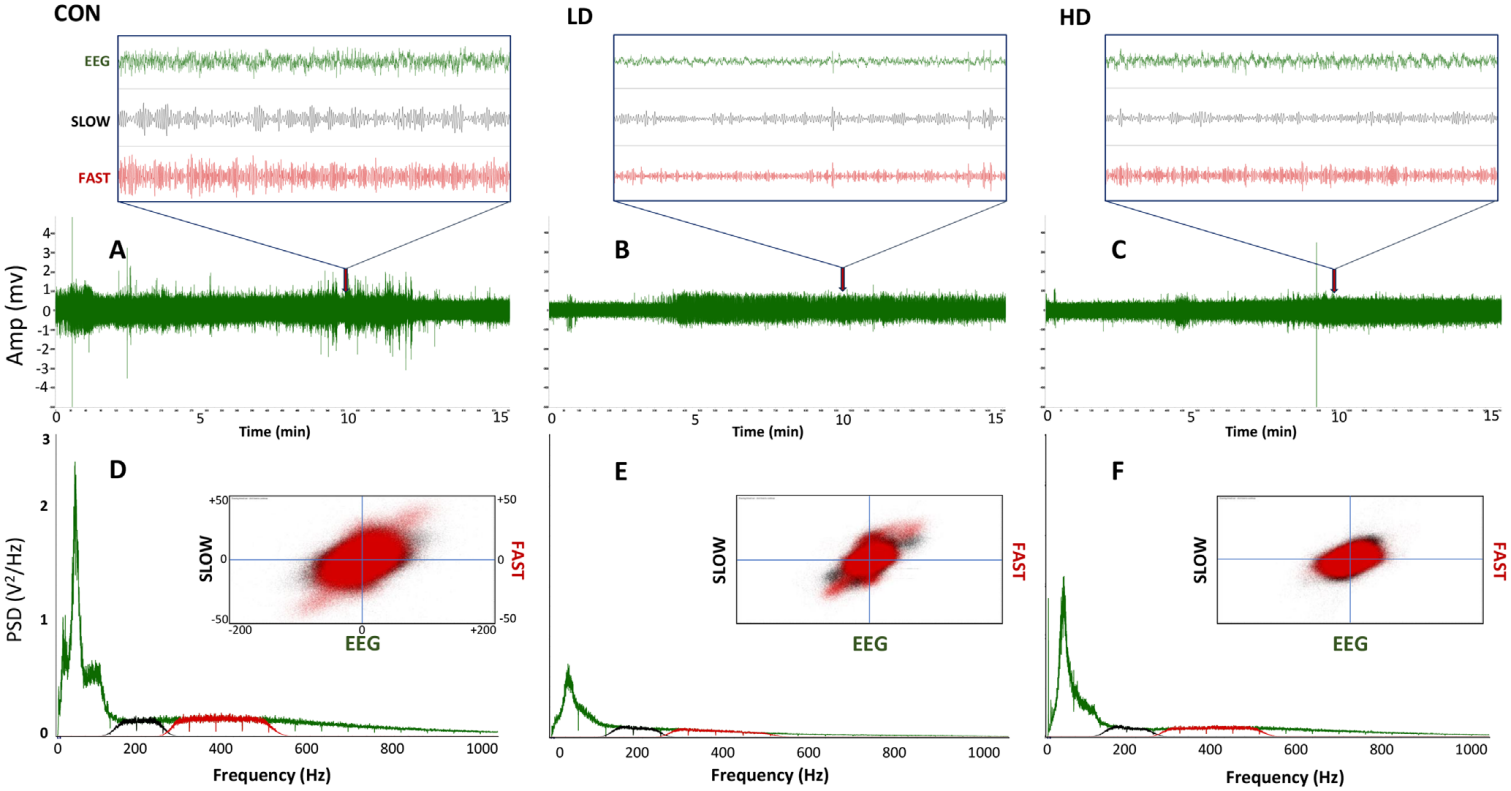
Comparative analysis of EEG activity across the control (CON), TNF low‐dose (LD), and TNF high‐dose (HD) groups. (A–C) EEG amplitude over a 15‐min interval for each condition. (A–C) Inset plots zoom into a selected 2‐s segment to highlight the differences in waveform morphology of EEG (green), along with isolated slow‐wave (black) and fast‐wave (red) components. (D–F) Power spectral density (PSD) plots for each treatment condition illustrate the spectral distribution across different frequencies. The peak shifts and power changes indicate alterations in brain wave activity in response to distinct TNF dosages. (D–F) Insets provide EEG feature space plots, showing the separation between the slow and the fast wave dynamics under each condition.

To gain a better understanding of TNF's effects on inhibitory neurotransmission, we utilized EEG recordings. Analysis of the EEG signals from the representative cortical recordings of individual mice revealed that the amplitude of field potentials decreases in TNF‐supplemented mice, while the EEG signals in supplemented animals are also more stable and less variable compared to control mice (Figure 5A–C). Compared to the control condition (A), the LD (B) and HD (C) groups exhibit reduced EEG amplitudes, particularly in the fast‐frequency range, indicating a dose‐dependent decrease in high‐frequency neural activity. The amplitudes of EEG traces beneath each panel confirm this pattern and show a significant decrease in signal intensity observed in response to TNF supplementation (Figure 5A–C). Power spectral density (PSD) plots (D–F) further support this observation. In all groups, power is primarily concentrated at lower frequencies (< 100 Hz), with a significant decrease in power in response to TNF supplementation (Figure 5D–F). Power spectral densities (PSDs) from brains of mice supplemented with TNF, LD (E) and HD (F) also show reduced power in higher frequency bands. The fast‐frequency components (red lines) display a clear downward shift in power with increasing dose.

The scatter plots show the relationship between the “slow” and the “fast” components of the EEG and provide a visual representation of how these components correlate with the EEG recordings under different treatment conditions. Inset scatterplots in Figure 5D–F show the distribution of EEG amplitudes against extracted slow and fast components. In the control group, EEG activity demonstrates a wider spread towards fast‐frequency activity. In contrast, the LD and HD groups cluster tightly around low values for both slow and fast frequencies. This suggests a robust association between treatment dose and decrease in high‐frequency neural dynamics.

These data demonstrate that TNF supplementation changes the dynamics of the EEG signals, which is consistent with the dose‐dependent decreases in the amplitudes of field potentials and the amplitudes of the fast and slow ripples observed in TNF‐supplemented mice, indicating that the treatment induces a dose‐dependent decrease of cortical excitability.

### 3.5 Thymus Extract Supplementation Decreases PSDs in a Dose‐Dependent Manner

**FIGURE 6:**
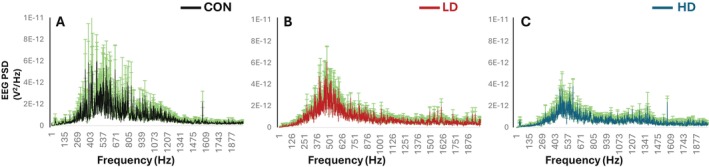
Comparison of PSDs of EEG signals obtained from control (CON) mice, and mice supplemented with TNF at a low dose (LD) or a high dose (HD), shows the distribution of power across a range of frequencies. (A) control group (*n* = 14, black); (B) LD group (*n* = 12, red); (C) HD group (*n* = 12, blue). Supplementation with TNF significantly reduced the power of PSDs at both—low and high doses (F[2,9.54E‐20] = 541.707; *p* < 0.001), with HD being most effective at reducing the overall PSD power compared to the other two conditions, with the PSD curve showing a flatter shape and lower peaks, especially in the lower frequency ranges. These results demonstrate a dose‐dependent decrease in EEG activity and spectral power in TNF‐supplemented animals.

The observed reduction in the amplitudes of field potentials and slow and fast ripples in TNF‐treated animals led us to examine PSDs in more detail. Analysis of the EEG signals obtained by pooling the data from the recordings obtained from each of the three animal groups (CON, LD and HD) revealed the power distribution of the EEG signals across a range of frequencies (1 Hz to 2000 Hz) (Figure 6A–C). We detected a noticeable peak around 500 Hz in all three conditions. Supplementation with TNF led to a significant decrease in PSD peak amplitude of the EEG signal in a dose‐dependent manner (F[2,9.54E‐20] = 541.707, *p* < 0.001) (Figure 6A vs. Figure 6B,C). These findings demonstrate that TNF exerts an inhibiting effect on brain excitability, leading to decreased electrical activity detected by the EEG recordings and the amplitudes of the PSDs. The overall power distribution across the frequency range shows that among the control, LD and HD mice, the HD group displays the lowest power peak, followed by LD animals (Figure 6A vs. Figure 6B,C). Supplementation with TNF at a high dose exerts a more pronounced effect on the EEG activity compared to the low dose (Figure 6B vs. Figure 6C). Together, the consistency of the frequency peaks and the dose‐dependent decrease in power demonstrate that TNF supplementation modulates brain activity and may potentially have an impact on brain function and behavior.

### 3.6 Thymus Extract Supplementation Shifts the Power of Low Frequency Oscillations

**FIGURE 7:**
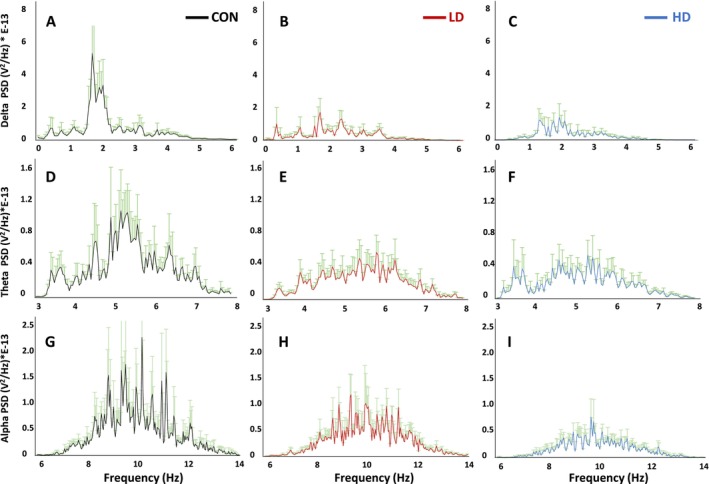
Power spectral density (PSD) analysis of EEG signals across three frequency bands—delta, theta and alpha—in control mice (CON, *n* = 14) and mice supplemented with TNF at a low dose (LD, *n* = 12) or a high dose (HD, *n* = 12). (A‐C) Delta wave PSD (0–6 Hz) exhibits a prominent peak in the control animals (A), indicative of significant delta wave activity, while delta activity is significantly attenuated in LD and HD groups compared to control animals (F[2,1.418E‐25] = 4.496; *p* < 0.01) (B and C). (D‐F) Theta band (3–8 Hz) PSD in the control group (D) displays a peak around 5 Hz and its amplitude is significantly reduced in LD and HD animals (F[2,8.884E‐20] = 34.789; *p* < 0.001) in a dose‐dependent manner (D vs. E, F). (G–I) Alpha band (8–14 Hz) PSD in control animals (G) shows multiple peaks within the alpha range, indicating robust alpha activity. LD and HD treatments (H and I) induce changes in alpha wave patterns, with overall lower peaks and shifts in peak frequency positions.

The aging brain undergoes many neurophysiological changes, including the decreases or increases in the spectral power of distinct types of neural oscillations, even in the absence of significant cognitive impairment [58, 59]. We therefore conducted a quantitative analysis of the PSD values obtained from the filtered field potential trace frequencies recorded from control and TNF‐supplemented mice. We detected decreases in PSD amplitudes and a shift of the frequency distribution of neuronal firing in the cortices of TNF‐supplemented mice (Figure 7). The low‐frequency oscillations (LFOs)—delta 0.4–4 Hz, theta 5–7 Hz, alpha 7–12 Hz—all displayed dose‐dependent decreases in peak amplitudes. In control animals, delta oscillations peak at approximately 2 Hz (Figure 7A). In mice supplemented with TNF at a low dose, a similar peak was observed around 2 Hz, but its power was significantly lower compared to control mice (*p* < 0.01; Figure 6B). In mice treated with TNF at a high dose, delta oscillations also peaked around 2 Hz, but with a significantly lower power compared to control animals (*p* < 0.01; Figure 7C). The theta wave peaked around 5–6 Hz in all conditions (Figure 7D–F), and its power was also reduced by TNF supplementation in a dose‐dependent manner. The alpha wave peaked around 10 Hz in all conditions (Figure 7G–I), and the reduction in the PSD peak amplitudes by TNF was similarly dose‐dependent, while the HD group exhibited a noticeable shift in the peak frequencies compared to both control and LD animals. These data demonstrate a pronounced dose‐dependent effect of TNF supplementation on EEG activity, with higher TNF dose being more potent at decreasing peak amplitudes of PSDs and inducing a shift in the peak frequencies. We also show that at a high dose, TNF increases the frequency power of the theta wave while decreasing the power of the delta wave, highlighting the diverse effects of TNF on neurophysiology.

### 3.7 The Power of Beta and Gamma Waves Is Decreased by TNF Supplementation in a Dose‐Dependent Manner

**FIGURE 8:**
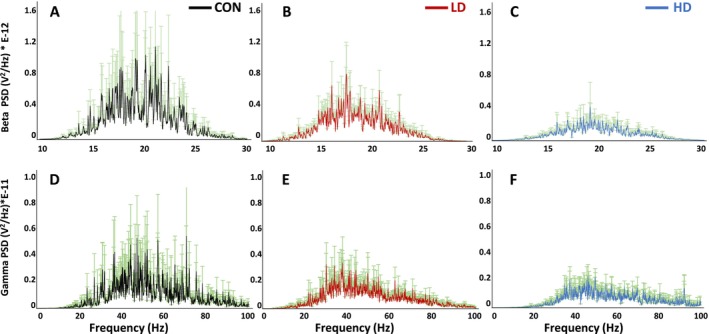
Power spectral densities (PSDs) of beta (top row) and gamma (bottom row) oscillations across different treatment conditions—control (CON, black), low dose (LD, red), and high dose (HD, blue). (A–C) Beta PSDs; (D–F) gamma PSDs. (A and D) PSDs for the control group (black) show prominent peaks in both beta (10–30 Hz) and gamma (30–100 Hz) frequencies. (B and E) PSDs in LD group animals (red) reveal reduced power of beta and gamma oscillations compared to control mice. (C and F) Power spectral densities (PSDs) in HD group animals (blue) show a significant decrease in power for both beta (*p* < 0.001) and gamma (*p* < 0.001) oscillations compared to CON group, especially at higher frequencies.

Cortical beta and gamma oscillations are believed to be generated by the circuits of GABAergic interneurons and involved in multiple cognitive processes, including attention, memory, and perception; disruption of these oscillations results is associated with cognitive dysfunction [60, 61, 62, 63, 64]. We found that the main frequency peaks for both beta and gamma bands were consistent across all three animals groups (CON, LD and HD)—around 20 Hz for beta (Figure 8A–C) and 40–60 Hz for gamma (Figure 8D–F). However, we detected a clear and significant dose‐dependent reduction in power for both beta (F[2,2.483E‐23] = 123.337; *p* < 0.001) and gamma waves (F[2,1.1998E‐25] = 220.657; *p* < 0.001) in LD and HD animal groups compared to control mice. These data suggest that TNF supplementation does not alter the frequency of the oscillations but primarily affects their power. Higher dose of TNF elicits greater reductions in PSDs, indicating that supplementation affects neural oscillations in a dose‐dependent manner.

### 3.8 Thymus Extract Suppresses the Peak Amplitudes of High Frequency Oscillations

**FIGURE 9:**
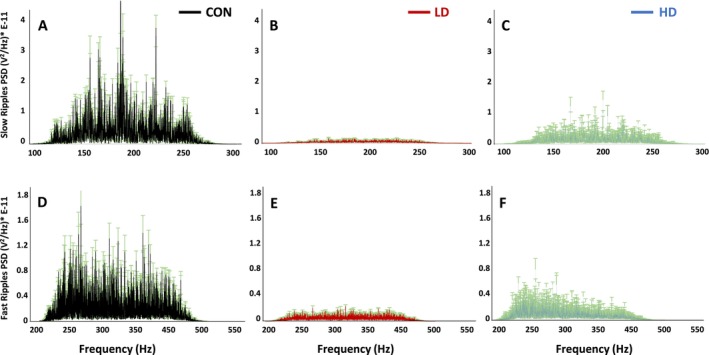
Power spectral densities (PSDs) of slow (top row) and fast (bottom row) ripples across different experimental conditions—control (CON, black), low dose (LD, red), and high dose (HD, blue). (A–C) Power spectral density (PSD) of slow ripples (100–300 Hz); (D‐F) PSD of fast ripples (200–500 Hz). (A and D) PSDs in control animals (black) show prominent peaks for both—the slow ripples (~100–250 Hz) and the fast ripples (~250–500 Hz). TNF supplementation had a significant effect on PSD amplitudes of both slow (F[2,5.118E‐20] = 455.218; *p* < 0.001) and fast (F[2,2.21E‐20] = 942.564; *p* < 0.001) ripple oscillations. (B and E) Power spectral densities (PSDs) in LD group animals (red) display significant suppression of both slow (*p* < 0.001) and fast (p < 0.001) ripple activity. (C and F) Power spectral densities (PSDs) in HD group animals (blue) show partial restoration of the ripple activity (fast and slow) compared to LD group, but remain significantly reduced compared to control group (*p* < 0.001).

We next examined high frequency oscillations, slow ripples at 125–250 Hz and fast ripples at 250–500 Hz, in control and TNF‐supplemented animals. We detected a significant decrease in the amplitudes of the slow and fast ripple PSDs in TNF‐supplemented mice compared to control animals (*p* < 0.001; Figure 9A–F). Control animals display the distinct peaks and the highest power in the slow and fast ripples among the three animal groups (Figure 9A,D vs. Figure 9B,C,E,F). Animals in the LD group displayed a significant reduction in power, indicating that TNF supplementation at low dose may suppress slow and fast ripple activity (Figure 9B,E). Animals in the HD group also display reduced power compared to the control mice, but higher power than observed in LD mice, suggesting a dose‐dependent supplementation effect, where high dose partially restores the slow and fast ripple activities (Figure 9C,F). These results demonstrate that TNF supplementation reduces both—slow and fast ripple activities—and suggest that the effect of the supplement on these types of neural oscillations may be complex. Together, our data indicate that the field potentials recorded from cortical neuronal circuits in TNF‐supplemented mice reflect reduced bursting activity, consistent with a state of reduced neuronal excitability.

## 4 Discussion

Many aspects of inhibitory neurotransmission are disrupted during aging and in neurodegenerative disease conditions, including the declining numbers of neurons that are able to produce GABA—the main inhibitory neurotransmitter in the CNS [11, 14, 19, 20, 21, 22], fewer presynaptic terminals that remain functional [65, 66], along with altered expression and/or function of various molecular components of the GABAergic system. Among these are reduced basal levels of GABA [6, 15], accompanied by a reduction in its release [11, 67], and changes in GABA_A_ receptor binding properties that are associated with reduced binding of GABA to its receptors [6, 68, 69, 70].

Lower basal levels of GABA in the aging brain and in neurologic disorders may be due to lower expression levels of the key enzymes responsible for its synthesis, GAD65 and GAD67, which were also shown to decrease significantly in the cortex and hippocampus in aged compared to young rodents [16, 17, 18, 52]. In this study, we found that GAD65 levels were significantly higher in aging mice supplemented with both low and high doses of TNF compared to control aging animals, while GAD67 was significantly increased only with a high dose TNF supplementation. A significant decline in GABA receptors in the brains of older individuals and older experimental animals was also reported [9, 13, 14, 15]. Similarly to GAD67, the levels of β2/β3 subunits of the GABA_A_ receptor were significantly increased only by a high dose of TNF. These findings suggest that a higher TNF dose used in this study is more potent at inducing the cellular or molecular changes necessary to support higher levels of key GABAergic system proteins in the aging mouse brain.

Thymus nuclear fraction (TNF) is an extract of a bovine thymus gland and is therefore likely to contain a mixture of thymic peptides that were previously isolated and characterized, albeit possibly at lower amounts compared to concentrated peptide preparations [71]. Some of the better studied thymic peptides and extracts were tested in models of neurologic diseases and were proposed to serve neuroprotective and anti‐inflammatory roles [44, 72, 73]. For instance, thymosin beta 4 (Tβ4) peptide was demonstrated to prevent cellular loss and enhance the functional recovery from TBI even when administered 6–24 h post injury [48, 74]. Interestingly, the neuroprotective function of Tβ4 was also found to be dose‐dependent, similarly to what we observed with TNF supplementation in this study. Given that thymic peptides can protect the brain from injury‐induced cell loss, there is a possibility that the increases in GAD65/67 and GABA_A_ β2/β3 subunits in response to TNF supplementation may reflect the attenuation of age‐related loss of cells that are a part of the inhibitory neurotransmission circuitry, thereby leading to preservation of GABAergic system protein expression.

Unlike Tβ4 and other thymic peptide preparations, however, which are delivered using injections, TNF is a nutritional supplement and is administered orally. As an extract of a complex thymus tissue matrix, TNF contains bioactive components that are likely missing from purified thymic peptide products. We recently demonstrated that TNF delivers appreciable amounts of bioavailable polyamines (PAs), such as spermidine, spermine, and putrescine, and can effectively elevate blood and tissue levels of spermidine and spermine in rats [75]. Even though PAs are thought to possess low blood–brain barrier (BBB) permeability, they can enter the brain in conditions that compromise BBB integrity, such as aging, inflammation, or injury, or through specialized transporters [76, 77]. Spermidine, one of the most abundant and bioactive PAs, is thought to regulate autophagy—a process that is vital for supporting cognitive function during aging and for longevity [78, 79, 80, 81, 82]. Importantly, spermidine was shown to improve cognitive function in animal models [78, 83, 84, 85], including aging mice [86].

Suggested neuroprotective roles of PAs, however, do not solely rely on the orchestration of the autophagy process but also on their additional pleiotropic biochemical activities, such as reducing oxidative and toxic stress by scavenging reactive oxygen species (ROS) and activating the nuclear factor erythroid 2‐related factor 2 (Nrf2) signaling pathway [87, 88, 89] regulating mitochondrial function, biogenesis and mitophagy [90, 91, 92], modulating neuroinflammation [93, 94] and regulating gene expression [95, 96]. Together, these diverse functions of PAs in supporting cellular homeostasis may be beneficial for neuronal survival during aging and hence—for the preservation of the inhibitory neurotransmission mediated by the GABAergic system neurons.

In the context of neurotransmission, PAs can modulate neuronal excitability in multiple ways. First, intracellular PAs can regulate the functions of glutamate receptors, such as *N*‐methyl‐d‐aspartate (NMDA), α‐amino‐3‐hydroxy‐5‐methyl‐4‐isoxazolepropionic acid (AMPA), and kainate (KA) receptors [97, 98, 99]. Depending on the concentrations of individual PAs and glutamatergic receptor type, the physiological effects of glutamate receptor modulation by PAs may vary, but can in fact include the reduction in neuronal excitability. Spermidine and spermine are the PAs that were shown to block inward rectifying potassium channels, some of which are used by GABA_B_ receptors during signal transduction and, thereby, can impact inhibitory neurotransmission [97, 99, 100]. Finally, in the rodent brain, putrescine was demonstrated to be metabolized as GABA in vivo [101, 102]. While GABA levels were not measured in this study, there is a possibility that putrescine delivered through TNF supplementation could contribute to the enhancement of inhibitory neurotransmission as well.

Consistent with the increases in the levels of GABAergic system proteins in response to TNF supplementation, cortical excitability in treated animals was reduced. While normal aging in mice was found to be accompanied by a global reduction in cortical neuronal excitability and spectral power [103], lower cortical excitability in humans is linked to better cognitive resilience during aging and a reduced risk of neurodegeneration [23, 104, 105, 106]. Similarly, the susceptibility of aging mice to kainate‐induced seizures may be coupled with increased neuronal excitability and diminished expression of AMPA, NMDA, KA, and GABA_A_ receptors, whose function and/or expression can be modulated by PAs, as demonstrated by others and in this study with GABA_A_ receptor β2/β3 subunit expression levels [97, 98, 99, 107].

The functional implications of TNF‐induced decreases in neuronal excitability have yet to be resolved. Nevertheless, our data demonstrate dose‐dependent and frequency‐specific effects of TNF on cortical ripples. Slow (∼100–150 Hz) and fast (∼250–500 Hz) ripples are distinctly altered by low versus high TNF doses: our findings suggest that low TNF dosing appears to subtly modulate the ripple activity, possibly compensating for or modulating the excitatory‐inhibitory balance, whereas high TNF doses consistently lead to a decrease in ripple power, particularly at slow ripple frequencies, compared to vehicle‐treated animals. This decrease could represent a decrease in array firing or failure to synchronize interneuron networks necessary for slow ripples. Nevertheless, the dose‐dependent decline in both ripple types suggests that TNF affects a number of ripple‐generating mechanisms, perhaps through changes in synaptic transmission, decreased pyramidal bursting, or decreased inhibitory control. The stronger suppression of slow ripples relative to fast ripples at higher doses may also indicate selective vulnerability of the circuits involved in lower‐frequency oscillations.

Given the well‐established role of ripples—particularly slow ripples—in memory consolidation and hippocampal‐cortical communication during rest and sleep, TNF‐induced reduction may have significant implications for cognitive function. Future studies will be necessary to determine whether the observed changes in ripple activity translate into behavioral deficits or altered mnemonic processing in TNF‐treated animals. However, we determined that locomotor activity in aging mice supplemented with TNF at a high dose is preserved despite the significant suppression of amplitudes of cortical oscillations across a range of frequency bands.

Moreover, sustained locomotor activity in mice supplemented with a high dose of TNF is accompanied by a trend towards reduction in animal body weights compared to control and low dose groups, which display age‐related weight gain that is known to occur in aging male C57BL/6 mice [108]. In addition to supporting higher activity levels, high doses of TNF may affect animal metabolism in a manner consistent with spermidine acting as a mimetic of caloric restriction [109, 110].

Together, our results demonstrate that TNF supplementation exerts a profound, dose‐dependent effect on neurotransmission in the aging mouse brain. Considering that sustained supplementation of rats with TNF prior to and post TBI leads to a significant improvement in animal functional recovery, the reduced neuronal excitability observed in this study may reflect the benefit of TNF supplementation in conditions where neuronal function or brain tissue integrity are compromised [43]. Future studies elucidating the mechanisms of TNF‐dependent regulation of neuronal excitability will determine its potential application in clinical practice.

## Author Contributions

The authors' responsibilities were as follows: A.E.‐I., B.F.E.‐K.: conceptualization; A.E.‐I., B.F.E.‐K.: methodology; A.E.‐I., B.F.E.‐K., N.S.: investigation; A.E.‐I: formal analysis; A.E.‐I., B.F.E.‐K., N.S.: visualization; A.E.‐I., N.S.: writing – original draft; A.E.‐I., N.S., B.F.E.‐K.: writing – review and editing; B.F.E.‐K.: resources; all authors: read and approved the final manuscript.

## Conflicts of Interest

N.S. and B.F.E.‐K. are salaried employees of Standard Process Inc.; all other authors declare no conflicts of interest.

## Data Availability

The data that support the findings of this study are either available in the Results section of this article, or available on request from the corresponding authors.
